# Publisher Correction: Neutral bots probe political bias on social media

**DOI:** 10.1038/s41467-021-27855-8

**Published:** 2022-01-05

**Authors:** Wen Chen, Diogo Pacheco, Kai-Cheng Yang, Filippo Menczer

**Affiliations:** 1 Observatory on Social Media, Indiana University, Bloomington, IN USA; 2 Department of Computer Science, University of Exeter, Exeter, UK

**Keywords:** Information technology, Technology, Communication, Politics, Society

Correction to: *Nature Communications* 10.1038/s41467-021-25738-6, published online 22 September 2021.

In the original HTML and PDF version of this Article, the phrase “et al.” was inadvertently missing from the reference list for references 3, 18, 33 and 35 and the hyperlink for reference 22 did not work. In addition, the repository names were missing for reference 60 and 65 (Harvard Dataverse and Zenodo, respectively). This has been corrected in the HTML and PDF versions of the Article.

In the original HTML version of the paper, the Article and ADS hyperlinks for reference 33 linked to the incorrect paper. These issues have now been corrected in the HTML version of the Article.

