# Supplementary material for: Neutral bots probe political bias on social media
Source: Nat Commun. 2021 Sep 22;12:5580. doi: 10.1038/s41467-021-25738-6 (PMC8458339; doi:10.1038/s41467-021-25738-6)
Supplement: Supplementary file 1 — Supplementary Information [file 41467_2021_25738_MOESM1_ESM.pdf]

Neutral bots probe political bias on social media

Wen Chen, Diogo Pacheco, Kai-Cheng Yang, and Filippo Menczer

Supplementary information

Supplementary methods

Seed accounts

While initializing the friend lists of the drifters, we aim to select five Twitter accounts that are associated with established, active, and popular U.S. news sources and that span the full range of the U.S. political spectrum. Supplementary Table 1 lists the ten news sources with most followers on Twitter in each political leaning bin. The political leaning of the sources in the table is obtained from the AllSides media bias rating list (`www.allsides.com/media-bias/media-bias-ratings`). Note that *The Wall Street Journal* is categorized as both center and center-right by the list. We assign it to the center-right category. The selected accounts (in bold) are among the most popular news sources on Twitter.

We also wish to verify that the seed accounts are popular among active Twitter users who are politically aligned with those sources. To this end, we started with a 10% random sample of public tweets on August 1, 2019, comprising about 36M tweets from 14M unique accounts. We sampled 500k of these accounts; for each of them, we calculated the bot score using the BotometerLite tool (Yang *et al.* 2020). We removed likely bots (those with bot score above 0.5) as well as non-English accounts, leaving 151,570 accounts. We extracted tweets by those accounts from the 10% random sample during one week around August 1, 2019. We used the links shared in those tweets to assign a political score to each of those accounts (see Methods). We filtered out accounts for which we could not assign a score. That left 26,304 accounts with a political score. We grouped these accounts into five political bins using thresholds -1, -0.5, -0.1, +0.1, +0.5, and +1, yielding groups of 712, 5,280, 11,422, 8,237, and 653 accounts in the left, center-left, center, center-right, and right bins, respectively. By examining the friends of these accounts, we eliminated those who did not follow any of the news sources in the AllSides list. 4,884 accounts remained: 187, 1,362, 1,623, 1,444, and 268 in the five groups, respectively. Finally, Supplementary Table 1 reports the proportions of accounts in each of these groups who follow the top sources in the AllSides list. This confirms that the seed sources are among the most followed by active Twitter users who are politically aligned with those sources.

Drifter actions and probabilities

An *action* is performed upon a sentence, an existing tweet, or a user. These inputs are selected from *sources* that are described below. A drifter can perform

Supplementary Table 1: **News sources with most followers on Twitter for each political bin.** Data collected on October 22, 2020. We also consider a sample of active accounts in each group and report the percentage who follow each source. Seed accounts used for initializing the friend lists of the drifters are highlighted in bold.

Leaning	News source	Twitter handle	Followers	
			Total	Within group
Left	HuffPost	`@HuffPost`	11.4M	34%
	The New Yorker	`@NewYorker`	8.9M	30%
	MSNBC	`@MSNBC`	3.6M	29%
	Mother Jones	`@MotherJones`	850.7k	29%
	Vox	`@voxdotcom`	983.6k	21%
	Slate	`@Slate`	1.8M	20%
	**The Nation**	`@thenation`	1.2M	20%
	The Daily Beast	`@thedailybeast`	1.3M	19%
	Newsweek	`@Newsweek`	3.4M	14%
	BuzzFeed News	`@BuzzFeedNews`	1.3M	13%
C. Left	The New York Times	`@nytimes`	47.5M	48%
	**The Washington Post**	`@washingtonpost`	16.4M	43%
	CNN News	`@CNN`	50.2M	38%
	The Guardian	`@guardian`	9.1M	33%
	The Economist	`@TheEconomist`	25.0M	29%
	TIME	`@TIME`	17.4M	28%
	ABC News	`@ABC`	15.9M	23%
	NBC News	`@NBCNews`	7.8M	23%
	CBS News	`@CBSNews`	7.7M	20%
	Bloomberg	`@business`	6.7M	17%
Center	Associated Press	`@AP`	14.4M	34%
	BBC News	`@BBCWorld`	29.3M	31%
	Reuters	`@Reuters`	22.4M	28%
	NPR News	`@NPR`	8.4M	24%
	The Hill	`@thehill`	3.9M	15%
	**USA Today**	`@USATODAY`	4.1M	14%
	Axios	`@axios`	494.4k	6%
	Real Clear Politics	`@RealClearNews`	183.5k	3%
	Christian Science Monitor	`@csmonitor`	79.1k	2%
C. Right	Fox News	`@FoxNews`	19.8M	30%
	**The Wall Street Journal**	`@WSJ`	18.1M	22%
	New York Post	`@nypost`	1.9M	18%
	The Epoch Times	`@EpochTimes`	319.0k	17%
	NewsMax	`@newsmax`	223.1k	11%
	Washington Examiner	`@dcexaminer`	272.9k	8%
	Market Watch	`@MarketWatch`	3.8M	5%
	The Washington Times	`@WashTimes`	406.4k	5%
	Reason	`@reason`	260.2k	3%
	The American Conservative	`@amconmag`	53.0k	1%
Right	One America News	`@OANN`	1.1M	68%
	**Breitbart News**	`@BreitbartNews`	1.5M	61%
	The Daily Caller	`@DailyCaller`	775.2k	44%
	The Blaze	`@theblaze`	762.2k	31%
	The Federalist	`@FDRLST`	277.0k	29%
	The Daily Wire	`@realDailyWire`	493.9k	23%
	National Review	`@NRO`	350.5k	20%
	Fox News Opinion	`@FoxNewsOpinion`	155.8k	10%
	CBN News	`@CBNNews`	148.9k	9%
	Daily Mail US	`@DailyMail`	309.4k	4%

the following actions:

- **Tweet** – post a sentence from *Random Quotes*, *Trends*, or *Home Timeline*. For *Trends*, the sentence is the text of the selected tweet. For *Home Timeline*, the sentence is obtained by concatenating a short phrase from a manually compiled list (e.g., "Wow!" or "Maybe so.") with the link of the selected tweet. This emulates a quoted tweet.

- **Retweet** – select a tweet from *Trends*, *Home Timeline*, or a list of *Tweets Liked by Friends*, and retweet it.

- **Like** – like a tweet selected in the same way as for a *Retweet*.

- **Reply** – reply to a tweet from the *Mention Timeline*. The reply is generated using the ChatterBot library (`chatterbot.readthedocs.io`). In case of failure, the reply is a random phrase from the precompiled list described above.

- **Follow** – select a user to follow from the list of *Followers*, *Friends of Friends*, users who posted *Tweets liked by Friends*, or users who posted tweets in the *Home Timeline*.

- **Unfollow** – select a user to unfollow from the latest 200 in the list of *Friends*.

Input elements for actions are selected from candidate lists that we call *sources*. The selection is random with uniform probability distribution unless otherwise explained below. Due to limitations of the Twitter APIs, we imitate some basic mechanisms offered by the platform, such as suggestions to follow friends of friends. Sources are defined as follows:

- **Random Quotes** – sentences obtained from a random quote API (`api.quotable.io/random`).

- **Mention Timeline** – the latest 10 tweets in the mention timeline. If the drifter replied to any mentions in the past, this source only considers subsequent tweets.

- **Friends of Friends** – the model randomly selects three friends of the drifter and requests their latest 5,000 friends, ignoring those that are already friends of the drifter. A new friend is selected from the combined list with probability proportional to the occurrences in the list, to favor friends of multiple friends.

- **Friends** – most recent 200 friends. The user is selected from this list at random, but older friends are more likely to be unfollowed. We implement this mechanism by ranking friends chronologically; the latest friend has rank one. The unfollow probability is proportional to the rank. The initial friend can never be unfollowed.

- **Followers** – most recent 200 followers.
- **Trends** – list obtained by randomly selecting three trending topics in the U.S. and fetching the top five tweets in each topic by the default ranking.
- **Tweets Liked by Friends** – start from the latest 15 tweets from the home timeline. Select a random subset of at most ten friends who posted these tweets. Select the three latest tweets liked by each of the selected friends, excluding any by the drifter itself. Select one tweet at random from this combined list. Depending on the selected action, the source can return the tweet itself (for **Retweet** or **Like**) or its author (for **Follow**).
- **Home Timeline** – the latest 15 tweets in the home timeline.

We list the probabilities used in the bot behavior model in Supplementary Table 2. The numbers are inferred from a random sample of Twitter users. If the Follow or Unfollow action is selected, a precondition check is triggered. If the Follow precondition is not met, the Unfollow action is performed and viceversa; the two checks cannot both fail. A new friend can only be followed if the number of friends is sufficiently small compared to the number of followers: less than the number of followers plus 113. A friend can only be unfollowed if the drifter has at least 50 friends.

Extraction of hashtags and links

We accessed tweets using the Twitter API. Links (URLs) and hashtags were extracted from `entities` metadata. Tweets longer than 140 characters are truncated; in these cases, we extracted links and hashtags from the `extended_entities` metadata except for 4% of the tweets, for which this retrieval process failed.

Supplementary Table 2: **Probabilities of actions and sources in the drifter bot behavior model.** The probabilities of the actions add up to one, and so do the conditional probabilities of the sources given each action.

Action	P(Action)	Source	P(Source|Action)
Reply	0.05	Mention Timeline	1.0
Tweet	0.15	Random Quotes	0.3
		Trends	0.3
		Home Timeline	0.4
Retweet<br>Like	0.1<br>0.35	Trends	0.1
		Home Timeline	0.6
		Tweets Liked by Friends	0.3
Follow	0.25	Home Timeline	0.2
		Tweets Liked by Friends	0.2
		Friends of Friends	0.5
		Followers	0.1
Unfollow	0.1	Friends	1.0

Many links are compressed using URL-shortening services. We expanded shortened links via HTTP HEAD requests using a heuristics based on the length of the URL (20 characters of less), allowing multiple redirects with a 10-second timeout.

Calibration of political alignment scores

We calibrated alignment scores so that positive scores mean right-leaning hashtags/links and negative scores mean left-leaning hashtags/links. To this end, we selected the news source account `@USATODAY` to have a zero alignment score. We used the 200 most recent tweets by `@USATODAY` in early June to calculate the raw center alignment score s_c. We obtained $s_c = 0.058$ and $s_c = -0.246$ for the link-based and hashtag-based approach, respectively. The political alignment scores are then calibrated by $s = \frac{1}{N}\sum_i^N (t_i - s_c)$, where t_i is the score for tweet i and N is the number of tweets across which the score is aggregated.

Statistical analyses

All t-tests in our analyses are two-sided. The main results in the paper are significant at the 0.05 level. In cases where we compare five groups of drifters, a Bonferroni correction for multiple comparisons can be applied by dividing the significance level by $\binom{5}{2} = 10$.

Supplementary notes

Comparisons of follower growth rates and confounding factors

There are multiple ways to test whether drifters in one group gain followers significantly faster than those in another group (Fig. 1). The method reported in the main text focuses on the daily follower growth for each drifter bot. We record the follower count on a daily basis in our experiment, with a few exceptions due to technical issues. We calculate the daily growth rate for any two consecutive observations of the follower count. We then combine the data points from each group and use t-tests to compare different groups (n between 373 and 389).

Here we report on analyses based on two additional methods. In the first, we first combine the raw observations (follower-date pairs) from the drifters within the same group and then combine them across two groups to be compared, using a dummy variable to distinguish them. Finally we apply linear regression to this combined data set with an extra interaction term between elapsed time and the dummy variable. The coefficient of the interaction term indicates whether the growth rates between the two groups are significantly different ($n = 782$, $p < 0.001$ comparing Left vs. Center and $n = 779$, $p < 0.001$ comparing Right vs. Left).

In the last method, we use linear regression to estimate the follower growth rate for each drifter. We then use a two-sided t-test to compare the estimated growth rates of two different groups ($d.f. = 4, t = 5.43, p = 0.006$ comparing Right vs. Center; $d.f. = 4, t = 2.71, p = 0.054$ comparing Left vs. Center; and

$d.f. = 4, t = 2.60, p = 0.060$ comparing Right vs. Left). While the p-values are affected by the degrees of freedom, all three methods yield consistent results.

The differences in influence among drifters could be affected by the popularity of the seed accounts. Supplementary Fig. 1 shows the correlation between drifter influence and two measures of popularity of their respective seed accounts. We find no significant correlation between the numbers of followers of drifters and seed accounts (Pearson's $r = 0.05$, $p = 0.850$). However, the drifter influence is correlated with the popularity of the seeds among active accounts with similar political alignment (Pearson's $r = 0.52$, $p = 0.049$).

Individual political trajectories

Starting with the political alignment estimations, Fig. 5 in the main text shows the aggregated scores. Next we provide the individual trajectory of the political alignment for each drifter. Full-resolution vector images of the plots are available in the data repository (`github.com/IUNetSci/DrifterBot`).

Supplementary Fig. 2 shows the results from the link-based approach and Supplementary Fig. 3 shows the results from the hashtag-based approach. We observe that the trajectories diverge in several examples, suggesting that the initial conditions do not limit the variability of the evolution. Supplementary Fig. 4 shows the news feed bias computed for each drifter with both methods. Note that two of the Right drifters were temporarily suspended by Twitter in mid-November 2019, and we neglected to reactivate them until the end of the experiment.

Political bias of news feed algorithm

Supplementary Table 3 shows the results of the analysis of bias in the platform's news feed. As discussed in the main text, most effects have small size and are not consistent across link- and hashtag-based methods.

Follow-back rates

Supplementary Fig. 5 plots the relative overlap between friends and followers of each drifter to examine the reciprocity of the links. We observe a higher follow-back rate for partisan drifters, and especially conservative ones.

Descriptive statistics of drifters

Finally, Supplementary Table 4 provides descriptive statistics of the drifters, including the number of friends and followers, number of tweets liked, number of tweets posted (including retweets), number of hashtags and links with alignment scores in posted tweets, and total number of actions taken.

Supplementary references

Yang, K. C., Varol, O., Hui, P. M., & Menczer, F. (2020). Scalable and generalizable social bot detection through data selection. In Proceedings of the AAAI Conference on Artificial Intelligence, Vol. 34, No. 01, pp. 1096-1103

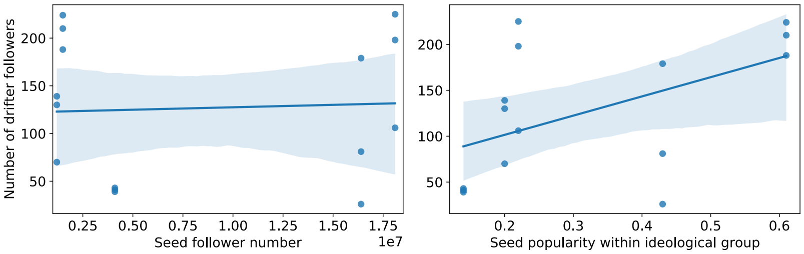

Supplementary Figure 1: **Confounding factors for drifter influence.** The scatter plots show the relationships between the number of followers of each drifter and (left) the number of followers and (right) the within-group popularity of its first friend. The number of followers of the drifters was measured on December 2, 2019. The seed popularity measures are taken from Supplementary Table 1; the methods to calculate overall and within-group popularity are documented in supplementary methods above. Shaded areas highlight the 95% confidence intervals around least-squared linear fits (solid lines). Source data are provided as a Source Data file.

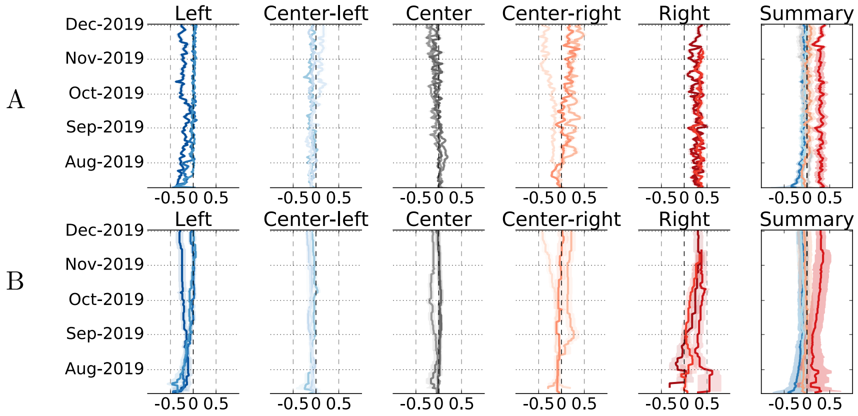

Supplementary Figure 2: **Political alignment timelines based on links for all fifteen bots.** A tweet is assigned a score between −1 (liberal) and +1 (conservative) based on the shared link domains. **A** Home timeline: daily average score of the last 50 tweets in the home timeline. **B** User timeline: daily average score of the last 20 tweets in the user timeline. The summary represents the average for each group. Colored confidence intervals indicate ±1 standard error. Source data are provided as a Source Data file.

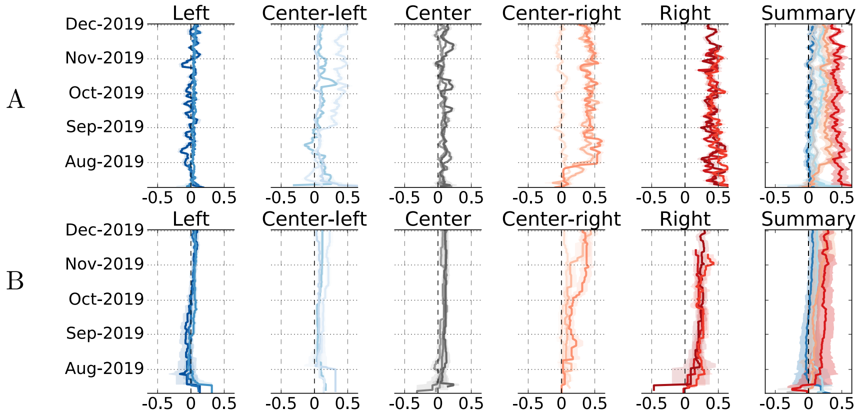

Supplementary Figure 3: **Political alignment timelines based on hashtags for all fifteen bots.** A tweet is assigned a score between −1 (liberal) and +1 (conservative) based on the shared hashtags. **A** Home timeline: daily average score of the last 50 tweets in the home timeline. **B** User timeline: daily average score of the last 20 tweets in the user timeline. The summary represents the average for each group. Colored confidence intervals indicate ±1 standard error. Source data are provided as a Source Data file.

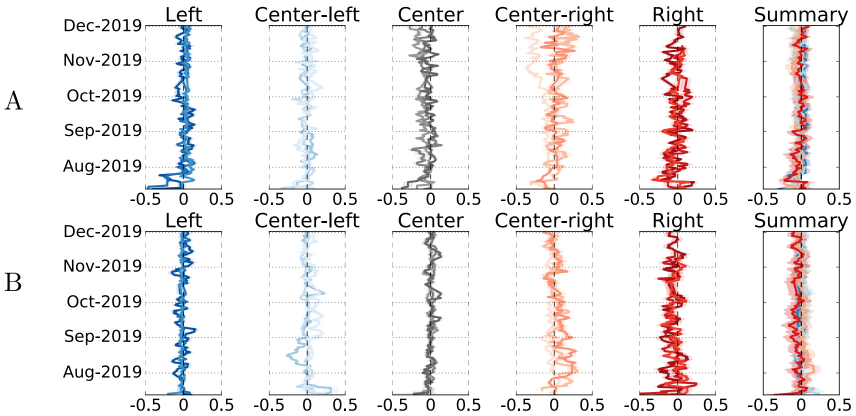

Supplementary Figure 4: **News feed bias for all fifteen bots.** Bias is measured by the difference in alignment between the account's home timeline and its friends' user timelines, based on **A** links and **B** hashtags. The summary represents the average for each group. Colored confidence intervals indicate ± 1 standard error. Source data are provided as a Source Data file.

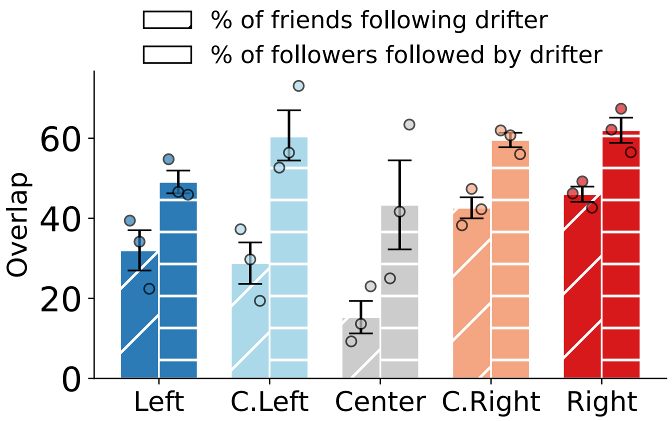

Supplementary Figure 5: Relative overlap between friends and followers of the drifters in each group. Error bars indicate standard errors ($n = 3$ drifters in each group). Source data are provided as a Source Data file.

Supplementary Table 3: **Political bias in the platform news feed.** Results of paired two-sided t-tests comparing political alignment scores of drifter home timelines and their friends' user timelines. Negative t values indicate left (liberal) bias, positive values indicate right (conservative) bias. Source data are provided as a Source Data file.

Drifters	Method	n	t	p	Cohen's d
Left	hashtag	387	-6.0	< 0.001	0.31
C. Left	hashtag	383	2.6	0.010	0.13
Center	hashtag	392	1.8	0.073	0.09
C. Right	hashtag	381	4.7	< 0.001	0.24
Right	hashtag	352	-10.6	< 0.001	0.56
Left	link	391	4.1	< 0.001	0.21
C. Left	link	389	-2.3	0.021	0.12
Center	link	393	-15.2	< 0.001	0.76
C. Right	link	385	-4.1	< 0.001	0.21
Right	link	352	-5.0	< 0.001	0.26

Supplementary Table 4: **Descriptive statistics of the drifters.** Data collected until 14 November 2019. Averages and standard deviations are shown for drifters in each group (highlighted) and across groups. Source data are provided as a Source Data file.

Drifters	Friends	Followers	Tweets	Likes	Hashtags	Links	Actions
Left	159	53	860	1218	169	275	3413
	230	118	841	1179	326	317	3357
	237	124	816	1177	339	281	3360
	209±35	98±32	839±18	1191±19	278±77	291±19	3377±26
C. Left	137	24	885	1258	213	300	3507
	269	150	837	1142	180	251	3237
	184	73	850	1233	143	257	3494
	197±55	82±52	857±20	1211±50	179±29	269±22	3413±124
Center	151	38	814	1117	171	232	3192
	152	38	827	1236	182	261	3446
	148	34	908	1195	158	265	3581
	150±2	37±2	850±42	1183±49	170±10	253±15	3406±161
C. Right	200	87	825	1205	171	273	3385
	291	177	779	1151	150	240	3254
	271	164	793	1108	154	233	3159
	254±39	143±40	799±19	1155±40	158±9	249±17	3266±93
Right	332	225	778	1051	203	210	3104
	322	211	816	1072	178	224	3107
	255	145	902	1256	179	249	3586
	303±34	194±35	832±52	1126±92	187±12	228±16	3266±227
All	223±64	111±65	835±39	1173±63	194±57	258±28	3345±157
