# Supplementary material for: Neutral bots probe political bias on social media
Source: Nat Commun. 2021 Sep 22;12:5580. doi: 10.1038/s41467-021-25738-6 (PMC8458339; doi:10.1038/s41467-021-25738-6)
Supplement: Supplementary file 3 — Reporting Summary [file 41467_2021_25738_MOESM3_ESM.pdf]

nature research

Corresponding author(s): Filippo Menczer

Last updated by author(s): May 10, 2021

Reporting Summary

Nature Research wishes to improve the reproducibility of the work that we publish. This form provides structure for consistency and transparency in reporting. For further information on Nature Research policies, see our Editorial Policies and the Editorial Policy Checklist.

Statistics

For all statistical analyses, confirm that the following items are present in the figure legend, table legend, main text, or Methods section.

n/a	Confirmed	
☐	☒	The exact sample size (n) for each experimental group/condition, given as a discrete number and unit of measurement
☐	☒	A statement on whether measurements were taken from distinct samples or whether the same sample was measured repeatedly
☐	☒	The statistical test(s) used AND whether they are one- or two-sided<br>*Only common tests should be described solely by name; describe more complex techniques in the Methods section.*
☐	☒	A description of all covariates tested
☒	☐	A description of any assumptions or corrections, such as tests of normality and adjustment for multiple comparisons
☐	☒	A full description of the statistical parameters including central tendency (e.g. means) or other basic estimates (e.g. regression coefficient) AND variation (e.g. standard deviation) or associated estimates of uncertainty (e.g. confidence intervals)
☐	☒	For null hypothesis testing, the test statistic (e.g. F, t, r) with confidence intervals, effect sizes, degrees of freedom and P value noted<br>*Give P values as exact values whenever suitable.*
☒	☐	For Bayesian analysis, information on the choice of priors and Markov chain Monte Carlo settings
☒	☐	For hierarchical and complex designs, identification of the appropriate level for tests and full reporting of outcomes
☐	☒	Estimates of effect sizes (e.g. Cohen's d, Pearson's r), indicating how they were calculated

Our web collection on statistics for biologists contains articles on many of the points above.

Software and code

Policy information about availability of computer code

Data collection	We provide all of our custom code for data collection in a public repository (github.com/IUNetSci/DrifterBot). The repository lists all dependencies on external libraries, such as twurl, chatterbot, and the Botometer Python client library.
Data analysis	We provide all of our custom code for data analysis in a public repository (github.com/IUNetSci/DrifterBot). We also use the gensim v.3.5.0 implementation of the word2vec algorithm (https://pypi.org/project/gensim/3.5.0/) and the Botometer Pro API (https://rapidapi.com/OSoMe/api/botometer-pro), including its BotometerLite endpoint.

For manuscripts utilizing custom algorithms or software that are central to the research but not yet described in published literature, software must be made available to editors and reviewers. We strongly encourage code deposition in a community repository (e.g. GitHub). See the Nature Research guidelines for submitting code & software for further information.

Data

Policy information about availability of data

All manuscripts must include a data availability statement. This statement should provide the following information, where applicable:

- Accession codes, unique identifiers, or web links for publicly available datasets
- A list of figures that have associated raw data
- A description of any restrictions on data availability

All of our data used to run the experiment and produce the figures in this paper is available in a public repository (github.com/IUNetSci/DrifterBot), to the extent that it is allowed by Twitter policies and IRB.

April 2020

Field-specific reporting

Please select the one below that is the best fit for your research. If you are not sure, read the appropriate sections before making your selection.

- [ ] Life sciences
- [x] Behavioural & social sciences
- [ ] Ecological, evolutionary & environmental sciences

For a reference copy of the document with all sections, see nature.com/documents/nr-reporting-summary-flat.pdf

Behavioural & social sciences study design

All studies must disclose on these points even when the disclosure is negative.

Study description	The study uses quantitative observational data based on analysis of information shared in public tweets. Some of the metrics are based on qualitative or mixed-method annotations provided by datasets in the literature.
Research sample	The main research sample are "social bots", automated Twitter accounts controlled by algorithms developed by the authors. These bots interact with Twitter accounts and the analyses also involve public posts from these Twitter accounts. We did not collect demographic information from these accounts; they may or may not be representative of the Twitter user population. The Twitter user population has been shown not to be representative of the general population, but was chosen because the study is focused on social media interactions, experiences, and biases.
Sampling strategy	Twitter accounts were recruited from the population of Twitter users through automated interactions with our bots: they followed our bots or were followed by the bots. No sample size was predetermined. The number of bots (15) was determined to minimize potential risks to the users (for example, the bots could share misinformation). This number was deemed sufficient based on preliminary analysis of the data showing significant differences between bots in different groups.
Data collection	Data was collected by custom software (available in our code repository) interacting with the Twitter API (application program interface) and was stored in a secure database for analysis. The data collection was automatic; the bots were activated autonomously by the software, as described in the manuscript. The researchers were not present and did not interfere with the software running the bots and collecting the data during the experiment. The data collected in the database was analyzed after the end of the experiment.
Timing	Data was collected between July 10, 2019 and December 1, 2019.
Data exclusions	No data was excluded.
Non-participation	No participant dropped out. Since the study involved observation of public behavior, a waiver of informed consent was granted per IU HRPP Policy on Informed Consent.
Randomization	Participants where grouped based on the bots with which they interacted, which differed based on political alignment of news sources first followed. The study controlled for this covariate by analyzing the groups distinctly.

Reporting for specific materials, systems and methods

We require information from authors about some types of materials, experimental systems and methods used in many studies. Here, indicate whether each material, system or method listed is relevant to your study. If you are not sure if a list item applies to your research, read the appropriate section before selecting a response.

Materials & experimental systems

n/a	Involved in the study	
x		Antibodies
x		Eukaryotic cell lines
x		Palaeontology and archaeology
x		Animals and other organisms
	x	Human research participants
x		Clinical data
x		Dual use research of concern

Methods

n/a	Involved in the study	
x		ChIP-seq
x		Flow cytometry
x		MRI-based neuroimaging

Human research participants

Policy information about studies involving human research participants

Population characteristics	See above.
Recruitment	See above. Some participants (the accounts who followed the bots) self-selected. Depending on the political alignment of the accounts with which a bot interacted, the bot would subsequently be exposed to different types of users and content, for example different amounts of social bots and misinformation. This was part of the political bias addressed in the study.
Ethics oversight	The study was approved by the Indiana University Human Subjects Committee (IRB Protocol #1905917253). Since the study involved observation of public behavior, a waiver of informed consent was granted per IU HRPP Policy on Informed Consent.

Note that full information on the approval of the study protocol must also be provided in the manuscript.
